# Tumor-Attentive Segmentation-Guided GAN for Synthesizing Breast Contrast-Enhanced MRI Without Contrast Agents

**DOI:** 10.1109/JTEHM.2022.3221918

**Published:** 2022-11-14

**Authors:** Eunjin Kim, Hwan-Ho Cho, Junmo Kwon, Young-Tack Oh, Eun Sook Ko, Hyunjin Park

**Affiliations:** Department of Electrical and Computer EngineeringSungkyunkwan University 35017 Suwon 16419 South Korea; Department of Medical Aritifical IntelligenceKonyang University 34966 Daejon 35365 South Korea; Samsung Medical CenterDepartment of Radiology, School of MedicineSungkyunkwan University 35017 Seoul 06351 South Korea; School of Electronic and Electrical EngineeringSungkyunkwan University 35017 Suwon 16419 South Korea; Center for Neuroscience Imaging ResearchInstitute for Basic Science 364806 Suwon 16419 South Korea

**Keywords:** Breast magnetic resonance imaging, image synthesis, tumor-attentive, segmentationguided, adversarial learning

## Abstract

Objective: Breast dynamic contrast-enhanced magnetic resonance imaging (DCE-MRI) is a sensitive imaging technique critical for breast cancer diagnosis. However, the administration of contrast agents poses a potential risk. This can be avoided if contrast-enhanced MRI can be obtained without using contrast agents. Thus, we aimed to generate T1-weighted contrast-enhanced MRI (ceT1) images from pre-contrast T1 weighted MRI (preT1) images in the breast. Methods: We proposed a generative adversarial network to synthesize ceT1 from preT1 breast images that adopted a local discriminator and segmentation task network to focus specifically on the tumor region in addition to the whole breast. The segmentation network performed a related task of segmentation of the tumor region, which allowed important tumor-related information to be enhanced. In addition, edge maps were included to provide explicit shape and structural information. Our approach was evaluated and compared with other methods in the local (n = 306) and external validation (n = 140) cohorts. Four evaluation metrics of normalized mean squared error (NRMSE), Pearson cross-correlation coefficients (CC), peak signal-to-noise ratio (PSNR), and structural similarity index map (SSIM) for the whole breast and tumor region were measured. An ablation study was performed to evaluate the incremental benefits of various components in our approach. Results: Our approach performed the best with an NRMSE 25.65, PSNR 54.80 dB, SSIM 0.91, and CC 0.88 on average, in the local test set. Conclusion: Performance gains were replicated in the validation cohort. Significance: We hope that our method will help patients avoid potentially harmful contrast agents. Clinical and Translational Impact Statement—Contrast agents are necessary to obtain DCE-MRI which is essential in breast cancer diagnosis. However, administration of contrast agents may cause side effects such as nephrogenic systemic fibrosis and risk of toxic residue deposits. Our approach can generate DCE-MRI without contrast agents using a generative deep neural network. Thus, our approach could help patients avoid potentially harmful contrast agents resulting in an improved diagnosis and treatment workflow for breast cancer.

## I. Introduction

Breast cancer is the most frequently diagnosed cancer and the second leading cause of cancer-related deaths in women worldwide [1]. Breast magnetic resonance imaging (MRI) is regarded as a sensitive imaging technique capable of diagnosing tumors [2]. Contrast agents (CAs) allow signal intensities to be enhanced, reflecting local metabolism, and using this mechanism, dynamic contrast-enhanced (DCE) MRI images can be obtained [3]. DCE MRI provides hemodynamic information and time-varying enhancing patterns of normal and diseased tissue types [4]. In particular, the patterns in the tumor region provide key information regarding diagnosis, prognosis prediction, and treatment planning [5]. However, the administration of gadolinium-based CAs may cause nephrogenic systemic fibrosis and risk of toxic residue deposits [6]. Moreover, there are cases in which CA injection or DCE MRI is restricted, such as in pregnant women or people with claustrophobia [7]. Therefore, a clinical need has emerged for a methodology capable of obtaining DCE MRI without CA.

Generative adversarial networks (GANs), a type of deep learning network, have drawn considerable attention for data synthesis. It consists of two networks: a generator that synthesizes new data along the distribution of training data and a discriminator that distinguishes the synthesized from authentic data [8]. The generator is trained to deceive the discriminator so that they compete with each other as a min–max game. Numerous studies have focused on image synthesis using GANs for various medical domains [9], [10], [11]. Nie et al. proposed a context-aware GAN on the brain and pelvic MRI and CT [12]. Yurt et al. suggested a multi-stream GAN for brain MR image synthesis using multimodal images [13]. In addition, the task-specific GAN has the potential to indirectly improve synthesis by learning the specified task in addition to the main synthesis task. Zhao et al. proposed a tripartite GAN for synthesizing liver contrast-enhanced MRI to improve tumor detection [14].

However, existing studies have focused on the synthesis of the entire images, not the tumor region that carries critical disease-related information [15], [16]. Tumor regions can be relatively small compared to the whole image, and thus, capturing the exquisite local characteristics of the tumor is challenging. Because of textural heterogeneity and diverse enhancing patterns in both normal and tumor regions of DCE MRI, for developing the synthesis network, we must pay careful attention to tumors. In addition, the traditional issue of blurring and learning instability of GANs could be more serious in the tumor region. Therefore, we need to pay more attention to generating the tumor region. Information about the tumor region could be better synthesized if a GAN network was supplemented with an auxiliary segmentation network (i.e., segmentor). This leads to the generation of an authentic-like tumor region while segmenting the tumor. The segmentor can provide fine-grained guidance on the enhancing pattern and texture of the tumor. In addition, we adopted a local discriminator to concentrate on the tumor region in addition to the regular discriminator on the whole image, motivated by existing works [17]. Edge maps contain important structural information, and many studies have utilized it to obtain more realistic images [18], [19]. This idea could help better capture fine tumor details in synthesizing breast MRI.

**FIGURE 1.**
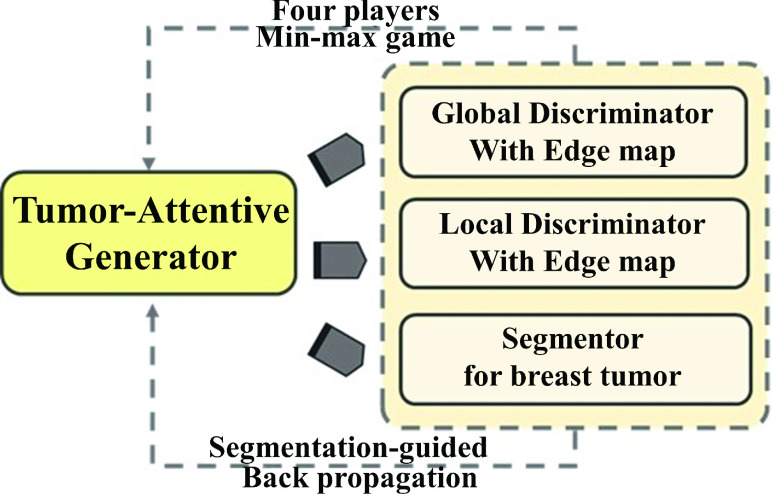
Schematic diagram of our proposed tumor-attentive segmentation-guided network. The tumor-attentive generator affects three network elements. In particular, the segmentor for breast tumor can guide the generator for the contrast-enhanced MRI in the tumor region.

The goal of this study was to generate realistic contrast-enhanced T1-weighted DCE MRI (i.e., ceT1) images from pre-contrast T1 weighted MRI of the breast. For our proposed approach, we adopted a local discriminator and segmentation network to focus specifically on the tumor region. In addition, edge maps were included to provide explicit structural information of the ceT1 images. Our approach was evaluated using both local and external test cohorts. A schematic diagram of the process is shown in Fig. 1.

## II. Materials and Methods

### A. Study Cohorts and Image Preprocessing

This study was a retrospective analysis of anonymized data and thus approval from the institutional review board was not required. The Samsung Medical Center (SMC) and Gil Hospital (GH) cohorts consisted of patients with breast cancer who had undergone surgery for invasive breast cancer between October 2011 and July 2012. We used breast DCE MRI of 306 and 140 patients from SMC and GH as the internal local cohort and external validation cohort, respectively. We included patients diagnosed with malignancy of invasive breast cancer and excluded patients treated with neoadjuvant chemotherapy. Details of the inclusion criteria are provided in the Appendix section (A).

For the SMC cohort, we collected DCE MRI images on a 1.5-Tesla or 3-Tesla scanner (Achieva, Philips Healthcare, Best, The Netherlands). For the GH cohort, MRI images were obtained using a 3-T scanner (Achieva, Philips Healthcare, Best, The Netherlands). The scan before contrast injection was noted as pre-contrast T1 (referred to as preT1). A scan of 1 min 30 s (early phase image referred to as ceT1) after injection was also acquired. We used preT1 (input) and ceT1 (target) images for our experiments. The early phase image was the target of the synthesis from a pre-contrast image. Because MRI scans had varying matrix sizes and slice thicknesses, we resampled them to isotropic resolution (1mm $\times1$mm $\times1$ mm) using B-splice interpolation. More details regarding DCE MRI are described in the Appendix section (B and Appendix Table 5). Breast tumor regions of interest (ROIs) were manually drawn around the entire visible tumor on the ceT1 images by a radiologist (ES, 15 years of experience) who was blinded to the clinical findings.

**TABLE 1:** Quantitative Evaluation for the Whole Breast Region and Tumor ROI of the Test Set. Bold Font Denotes the Best Performance Among Comparison. Each Cell Entry Has an Average Value Followed by Standard Deviation in Parentheses. * Denotes p < 0.001 by Permutation Tests

	Whole Breast Region			
	NRMSE( $\downarrow$)	PSNR( $\uparrow$)	SSIM( $\uparrow$)	CC( $\uparrow$)
Pix2Pix	31.681 (2.443)*	50.713 (1.489)*	0.863 (0.028)*	0.820 (0.036)*
ResGAN	30.884 (3.042)*	51.289 (1.934)*	0.865 (0.030)*	0.822 (0.038)*
EaGAN	28.788 (3.764)*	52.611 (2.070)*	0.876 (0.033)*	0.850 (0.031)*
TSGAN-Box	26.117 (2.358)*	52.669 (2.190)*	0.896 (0.026)*	0.898 (0.025)*
TSGAN	25.654 (2.626)	54.804 (2.544)	0.905 (0.024)	0.875 (0.029)
	Tumor ROI			
Pix2Pix	29.904 (4.314)*	71.541 (3.180)*	0.808 (0.065)*	0.796 (0.079)*
ResGAN	28.971 (4.509)*	72.240 (3.514)*	0.824 (0.070)*	0.811 (0.080)*
EaGAN	26.351 (4.534)*	72.897 (4.074)*	0.839 (0.064)*	0.838 (0.070)*
TSGAN-Box	25.024 (5.030)	73.409 (4.512)*	0.854 (0.065)	0.858 (0.069)
TSGAN	25.306 (4.814)	74.372 (4.312)	0.860 (0.063)	0.854 (0.069)

**TABLE 2:** Quantitative Evaluation for the Whole Breast Region and Tumor ROI of the External Validation Cohort. Bold Font Denotes the Best Performance Among Comparison. Each Cell Entry has an Average Value Followed by Standard Deviation in Parentheses. * Denotes p < 0.001 by Permutation Tests

	Whole Breast Region			
	NRMSE( $\downarrow$)	PSNR( $\uparrow$)	SSIM( $\uparrow$)	CC( $\uparrow$)
Pix2Pix	34.711 (2.355)*	48.875 (1.666)*	0.795 (0.031)*	0.788 (0.044)*
ResGAN	30.013 (6.278)*	52.561 (2.867)*	0.836 (0.044)*	0.844 (0.036)*
EaGAN	31.647 (3.705)*	50.075 (2.053)*	0.838 (0.036)*	0.840 (0.038)*
TSGAN-Box	25.496 (2.748)*	52.675 (1.927)*	0.873 (0.032)*	0.867 (0.036)*
TSGAN	21.249 (2.141)	56.619 (2.445)	0.897 (0.025)	0.888 (0.030)
	Tumor ROI			
Pix2Pix	28.306 (3.727)*	68.439 (3.288)*	0.774 (0.063)*	0.776 (0.068)*
ResGAN	33.561 (8.067)*	66.371 (3.319)*	0.747 (0.093)*	0.748 (0.103)*
EaGAN	30.502 (5.584)*	67.007 (3.342)*	0.760 (0.085)*	0.764 (0.093)*
TSGAN-Box	25.658 (4.358)*	69.227 (3.670)*	0.808 (0.076)*	0.819 (0.079)*
TSGAN	23.567 (4.403)	70.947 (4.481)	0.832 (0.074)	0.839 (0.073)

**TABLE 3:** Validity Assessment of the Independent Segmentation Task. Each Cell Entry Has an Average Value Followed by Standard Deviation in Parentheses

Training image	CeT1			CeT1 with Synthetic ceT1
Testing image	PreT1	Synthetic ceT1	CeT1	CeT1
BCE( $\downarrow$)	0.078 (0.038)	0.044 (0.027)	0.040 (0.022)	0.038 (0.024)
DSC( $\uparrow$)	0.166 (0.258)	0.622 (0.270)	0.656 (0.237)	0.681 (0.253)
ASSD( $\downarrow$)	43.177 (41.417)	10.74 (30.953)	2.551 (1.519)	2.512 (2.487)

**TABLE 4:** Performance Gains Achieved by Adding Various Components of Our Model. Each Cell Entry Has an Average Value Followed by Standard Deviation in Parentheses

	NRMSE( $\downarrow$)	PSNR( $\uparrow$)	SSIM( $\uparrow$)	CC( $\uparrow$)
Pix2Pix	31.681 (2.443)	50.713 (1.489)	0.863 (0.028)	0.820 (0.036)
ResGAN	30.884 (3.042)	51.289 (1.934)	0.865 (0.030)	0.822 (0.038)
+SN^a^	29.522 (2.312)	53.218 (2.017)	0.869 (0.029)	0.827 (0.036)
+LD	30.502 (3.081)	52.491 (2.275)	0.878 (0.028)	0.843 (0.035)
+Edge map	25.501 (2.484)	53.048 (2.162)	0.8999 (0.0271)	0.878 (0.030)
+Segmentor (Proposed)	25.654 (2.626)	54.804 (2.544)	0.905 (0.024)	0.875 (0.029)

**TABLE 5:** Imaging Parameters of the Cohorts

	SMC on 1.5 T	SMC on 3T	GH on 3T
TR(ms)	6.5	5.5	5.5
TE(ms)	2.5	2.8	2.8
Flip angle(°)	10	12	18
FOV (cm)	32 × 32	30 × 30	34 × 34
Matrix	376 × 374	500 × 237	424 × 424
Slice thickness (mm)	1.5	3	3
Imaging time after CA injection(s)	30, 90, 150, 210, 270, 330	30, 90, 150, 210, 270, 330	90, 180, 270, 360, 480

By random sampling for the internal cohort, we considered 2,233 slices as the training set using only the slices containing the tumor ROI from 230 subjects. The validation and test sets had 180 slices from 21 subjects and 583 slices from 55 subjects, respectively. All images were resized to 256 $\times256$ pixels. DCE MRI has a well-defined tumor-enhancing hemodynamic pattern, and if we apply a standard normalization per image, the enhancing patterns across different phases might be removed. Therefore, preT1 and ceT1 image intensities were clipped to the maximum in the ceT1 images in the training set and then linearly scaled to a range from −1 to 1. Flip-up and flip-down augmentations were randomly performed.

For the external validation cohort, we used 1,720 slices of DCE MR images using only the slices containing the tumor ROI and the preT1 image as input and the early phase image as the target. Images were resized to 256 $\times256$, and the same normalization was applied using the maximum of ceT1 in the validation cohort.

### B. TUMOR-Attentive Segmentation-Guided Generative Adversarial Network

**FIGURE 2.**
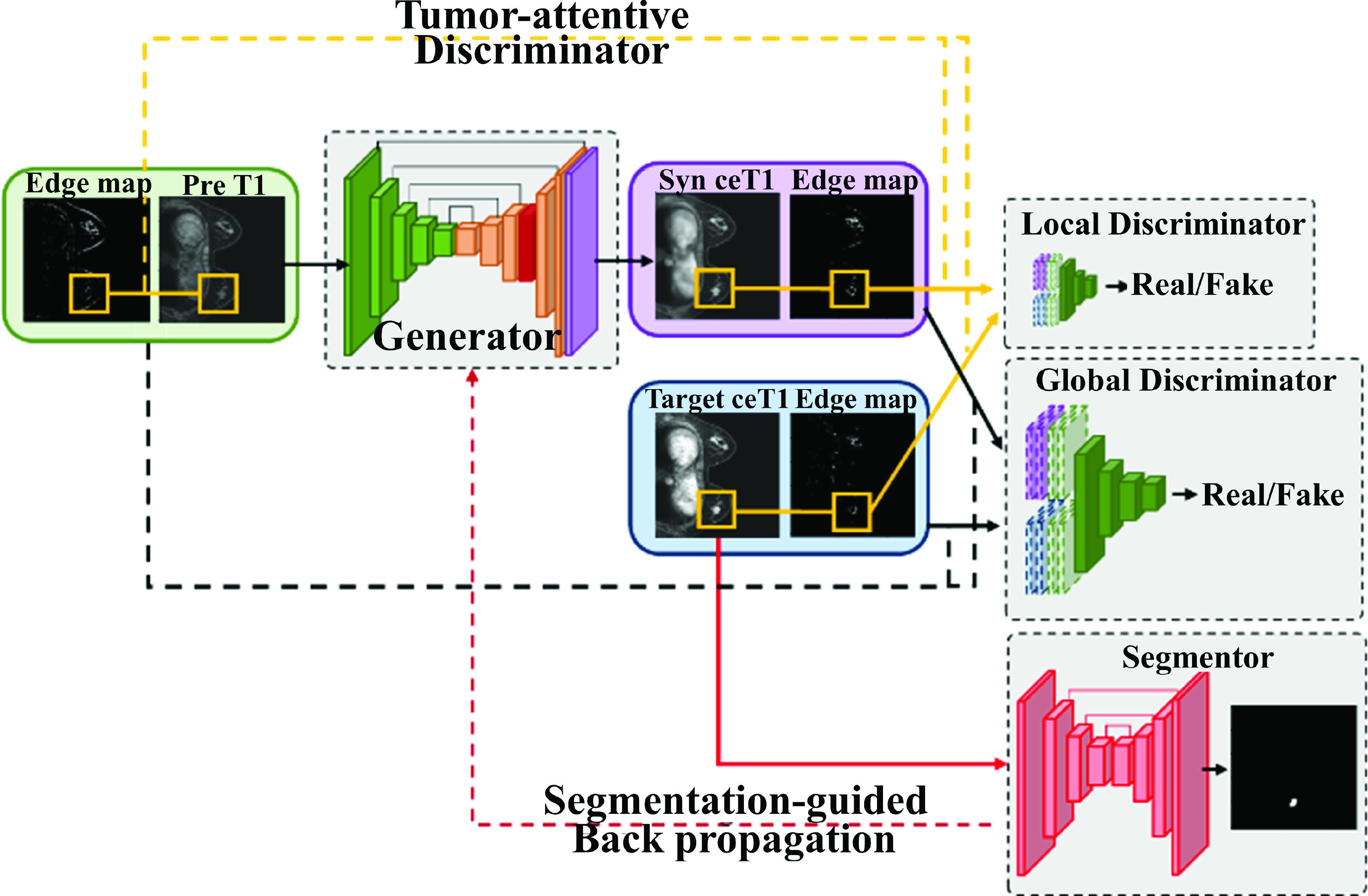
Overview of the TSGAN. The generator takes the input image (“preT1”) and outputs the synthesized ceT1 image (“Synthesized ceT1”). The target image (“ceT1”) and its edge map constitute a pair of images. Synthesized ceT1 and its edge map constitute another pair. The global discriminator takes the two pairs (the preT1 and real ceT1 pair or the preT1 and synthesized ceT1 pair) as input and discriminates which pair is the real or synthesized pair (“fake”). The LD does the same using tumor-centered $64\times 64$ patches. The segmentor takes the target ceT1 as input and trains the segmentation of the breast tumor. Synthesized ceT1 also is segmented, thus guiding the generator to resemble the target ceT1 tumor image. These three networks induce the generator to synthesize more authentic ceT1, focusing on the tumor region by backpropagation.

We designed a tumor-attentive segmentation-guided generative adversarial network (TSGAN) to synthesize a ceT1 image from a preT1 image. Fig. 2 shows the overall network structure of the proposed approach. The global discriminator learned the characteristics of time-varying contrast-enhancing patterns of whole breast image, and the local discriminator (LD) learned the details of contrast-enhanced tumor regions of the ceT1 image. They allowed the generator to synthesize more real-like ceT1 images in adversarial learning. In particular, the LD part made up the tumor-attentive aspect of our network. The segmentation network performed segmentation of the breast tumor, which encouraged important tumor-related information to be passed to the generator. This part was the segmentation-guided aspect of our network to focus on the tumor. The edge maps of the target ceT1 and synthesized ceT1 provided explicit morphological features to both discriminators, allowing the generators to map translations from preT1 to ceT1 images. In addition, an attention module was added to the middle part of the decoding steps of the generator. Residual learning and spectral normalization were adopted for the convolutional layers of the GAN for stable training.

#### 1) Generator

**FIGURE 3.**
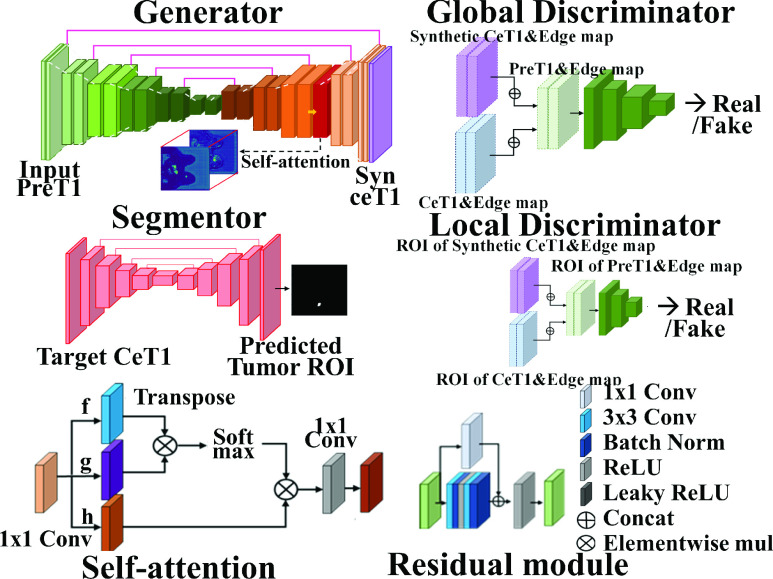
Network details. The upper left box included the generator structure using a modified U-net, residual block, and self-attention module. Each layer of the generator has the residual module before the down/up sampling. The bottom left box described the structure of the self-attention and residual module. Each of the three boxes on the right side indicated the segmentor, global discriminator, and LD.

We adopted U-Net as the backbone for the generator [20]. To achieve stable loss convergence, we constructed a generator with residual modules. The residual module trained the self-residuals after convolutional operations to achieve stable learning [21]. The down-sampling structure after the residual block contained $2\times2$ convolutional layers with stride two, batch normalization, and leaky ReLu. The procedure was stacked four times. In the decoding step, the up-sampling structure that included $2\times2$ convolutional transpose layers with stride two, batch normalization, and a ReLU activation layer passed through the residual module by concatenating with the shortcut connection. This procedure was repeated four times. To encourage the representation of important features, a self-attention module was inserted after the third sampling layer, which had 64 $\times64$ feature maps [22]. The module focused on semantic attribution, which is important for mapping from preT1 to ceT1, including enhancing patterns and the differences between them. We used spectral normalization (SN) to every up/down sampling structure, which prevented the weights from drastic gradient changes [23]. We applied SN to both discriminators as well as the generator and sought more stable convergence. Fig. 3 shows the details of the network.

#### 2) Global Discriminator

A global discriminator (GD) distinguished which pair of images was the target or was synthesized using the entire field of view (FOV). It consisted of four convolutional layers with stride 2 for a wide 32 $\times32$ receptive field with global dependencies constrained by the SN. It took four channels as input, composed of two pairs of images. One pair consisted of the entire ceT1 image and the corresponding edge map (ceT1 can be synthesized or targeted). The other pair was the preT1 image and its corresponding edge map. Using the preT1 pair as a condition, the discriminator had the discriminative power to find a ceT1 that was more compatible with the preT1 image.

The addition of an edge map explicitly exploited important structural features, which revealed the saliency of the enhancing pattern. Edge maps were computed by the normalized sum of the magnitudes of the Sobel filters applied in the horizontal and vertical directions. Edge map values were binarized using a threshold of 0.4. Training of GD allowed the generator to learn not only the intensity changes from the whole preT1 image to the entire ceT1 image but also the edge map-related changes in adversarial learning.

The feature map before the last convolution layer was used for feature matching [24]. The generator was forced to synthesize ceT1 images to have intermediate feature maps similar to those of the target ceT1 image and the synthetic ceT1 image.

#### 3) Local Discriminator

We developed our TSGAN that can discriminate the tumor region in more detail by attaching an LD. Because the FOV in LD was smaller than that in the whole breast image, the LD was constructed to have three shallow layers. The LD learned to discriminate not only whether the local tumor region was the target or fake but also whether the edge of the tumor region was the target or fake for preT1 as a condition. LD also took two pairs of images as input, as described for GD above. However, the pairs of ceT1 images and edge maps used for LD were cropped to 64 $\times64$ patches based on the center of the tumor.

The tumor edge maps allowed us to capture detailed contrast-enhancing tumor-specific properties that are critical in tumor synthesis. Like GD, LD used a feature matching method that allowed the generator to synthesize a realistic tumor in the feature space.

#### 4) Segmentor

**FIGURE 4.**
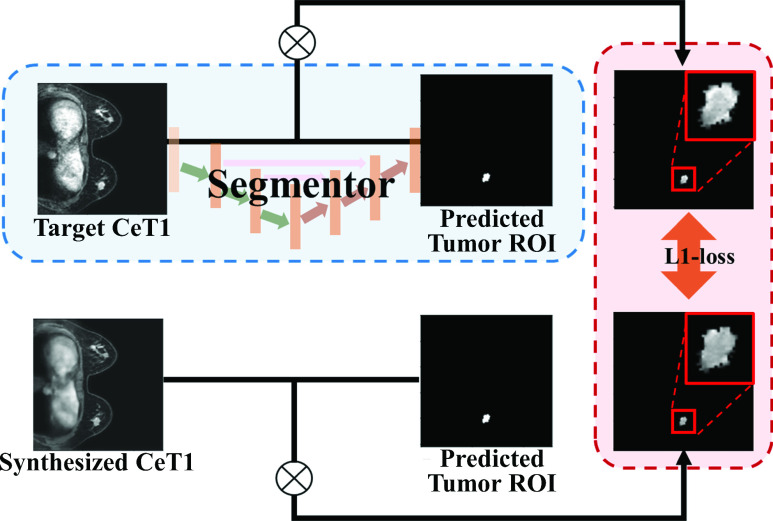
The segmentor was trained using the target ceT1 image as shown in the blue box. After multiplying the target/synthesized ceT1 image and the tumor ROIs predicted by the segmentor, tumor-related guidance was provided to the generator through L1-loss, as shown in the red box.

The segmentation network (i.e., segmentor) was trained with the other three networks simultaneously to synthesize the tumor ROI of the ceT1 image better. The segmentor was constructed using a standard U-Net backbone with skip connections and five down/up sampling layers without SN constraints. The down-sampling block consisted of a $2\times2$ convolutional layer with strides of 2, batch normalization, and a leaky ReLU activation layer. The up-sampling block performed the same except that convolutional transpose layers were used. The segmentor was trained using only the target ceT1 images to provide correct tumor guidance. The segmentor took the target ceT1 and synthesized ceT1 images as input and predicted two tumor ROIs from them. Each ROI was multiplied with the corresponding input ceT1 image, which contained the internal characteristics of the tumor, including texture and shape. The tumor ROI from the synthesized image was used to follow the distribution of the tumor ROI from the target image by transmitting the L1 loss to the generator. This allowed the generator to focus more on the tumor region. Fig. 4 shows the details of the segmentation network. Integrating these four parts, the overall structure was complete, focusing on the tumor ROI.

### C. Loss Function

We defined the three networks by minimizing the standard hinge loss among them i.e., generator (G), GD, and LD [25]. The segmentor (S) was defined by minimizing the combined loss of the dice loss with the binary cross-entropy. As described previously, we computed the edge maps for both target ceT1 and synthetic ceT1. Equation (1) was defined such that the GD distinguishes a pair of x (preT1 image and corresponding edge map) and y (target ceT1 image and corresponding edge map) as “real” and a pair of x (preT1 image and corresponding edge map) and G(x) (synthesized ceT1 image and corresponding edge map) as “fake.” Equation (2) is similar to (1) but focuses on the tumor ROI. In (1) and (2), G receives an x (preT1 image) and tries to generate a realistic ceT1 image and fool the GD and LD in adversarial learning:

\begin{align*}&\hspace {-1pc}L\left ({G,GD }\right) \\=&-E_{\left ({x,y }\right)\sim pdata} \\&\hspace {0.5pc}\,\left [{ \min \left ({0,-1+GD\left ({x,y,x_{edge},y_{edge} }\right) }\right) }\right]-E_{\left ({x,y }\right)\sim pdata} \\&\hspace {0.5pc}\,\left [{ \min \left ({0,-1-GD\left ({x,G\left ({x }\right),x_{edge},{G\left ({x }\right)}_{edge} }\right) }\right) }\right]. \tag{1}\\&\hspace {-1pc}L\left ({G,LD }\right) \\=&{-E}_{\left ({x,y }\right)\sim pROI}\left [{ \mathrm {min}(0,-1+LD(x,y,x_{edge},y_{edge}) }\right] \\&-\,E_{(x,y)\sim pROI} \\&\hspace {0.75pc}\,\left [{ \mathrm {min}(0,-1-LD(x,G\left ({x }\right),x_{edge},{G(x)}_{edge})) }\right].\tag{2}\end{align*}

Equation (3) describes the loss for the segmentor including the binary cross-entropy and dice loss commonly used in segmentation tasks by applying the target breast tumor ROI label as l and predicted label as $\tilde {\textrm {l}}$:

\begin{equation*} L(S)= {\textit{BCE}}(l,\tilde {l}) + {\textit{Dice}}(l,\tilde {l}).\tag{3}\end{equation*}

Equation (4) was defined as G learning to synthesize a ceT1 image to be close to y from various sources with the L1-norm. It consists of three terms for the entire image and the other three terms for the local tumor area. The generator’s L1 losses from the global image included the similarity between the entire FOV images, their edge maps, and the feature maps extracted just before the last convolution layer of the global discriminator ( ${GD}_{fm}$). This feature map had a size of $16\times16$ and a receptive field of 16 $\times16$ which reflected more local latent features than the output of the GD, which had a size of $8\times8$ and receptive field of 32 $\times32$. The generator was trained to make the feature maps extracted from the target ceT1 and synthesized ceT1 images to be similar in the latent space using feature matching.

With regard to the local region, the generator’s L1 losses were applied to the local tumor region masked with the predicted ROI. They included the similarity between the tumor ROI images, segmented ceT1 images, and feature maps extracted from the LD ( ${LD}_{fm}$). Similarly, in LD, feature maps extracted just before the last convolutional layer helped the generator to train better.

We intended to generate the whole breast in the earlier stages and focused on the local tumor region in the later stages. Therefore, we adopted curriculum learning, which gradually increases the weights according to the epoch [26]. This helped our complex model learn stably in the desired direction. The hyperparameters were tuned using the validation set. The weights ( $\lambda _{global}$, $\lambda _{edge}$, $\lambda _{curr}$) acted as hyperparameters and were empirically determined. The weights ( $\lambda _{global}$, $\lambda _{edge}$) of L1 regularization for the similarity of the whole breast ceT1 image and the corresponding edge map were set as 300 and 20 in the generator, respectively. The curriculum lambda ( $\mathrm {\lambda }_{curr}$) was five times the number of epochs before epoch 20 and remained at 100 afterward. The weights for the adversarial loss and feature matching from the two discriminators were set to 5 and 1. Equation (5) shows our final objective, including the weights ( $\lambda$) of G, as follows:

\begin{align*}&\hspace {-2pc}L_{L1}\left ({G }\right) \\=&\lambda _{global}E_{(x,y)\sim pdata}\left [{ {\vert \vert y-G(x)\vert \vert }_{1} }\right] \\&+\,\lambda _{edge}E_{(x,y)\sim pdata}\left [{ {\vert \vert y_{edge}-{G(x)}_{edge}\vert \vert }_{1} }\right] \\&+\,\lambda _{curr}E_{(x,y)\sim pROI}\left [{ {\vert \vert y-G(x)\vert \vert }_{1} }\right] \\&+\,\lambda _{curr}E_{(x,y)\sim pdata}\left [{ {\vert \vert S(y)\cdot y-S(G(x))\cdot G(x)\vert \vert }_{1} }\right] \\&+\,\lambda _{edge}E_{\left ({x,y }\right)\sim pROI}\left [{ {\vert \vert y_{edge}-{G(x)}_{edge}\vert \vert }_{1} }\right] \\&+\,E_{\left ({x,y }\right)\sim pdata}\left [{ {\vert \vert {GD}_{fm}(y)-{GD}_{fm}(G\left ({x }\right))\vert \vert }_{1} }\right] \\&+\,E_{\left ({x,y }\right)\sim pROI}\left [{ {\vert \vert {LD}_{fm}(y)-{LD}_{fm}(G\left ({x }\right))\vert \vert }_{1} }\right]. \tag{4}\\&\hspace {-2pc}Arg{min}_{G,S}~{max}_{GD,LD}L\left ({G,GD }\right)+L\left ({G,LD }\right) \\&+\,\lambda L_{L1}\left ({G }\right)+L\left ({S }\right).\tag{5}\end{align*}

### D. Evaluation

We trained the Pix2Pix network as the baseline GAN model to compare with our model [27]. We also trained well-established networks—the residual GAN (ResGAN), and edge-aware GAN (EaGAN)—as other baseline methods. ResGAN and EaGAN were implemented with residual modules and edge maps [18]. One additional model, TSGAN-Box, where the pixel-level semantic segmentation network was replaced with a detection network predicting the bounding box enclosing the tumor was trained using the same U-Net backbone. This was implemented to compare different mechanisms of tumor attention.

We performed quantitative analysis using four evaluation metrics for not only the whole breast region, but also the tumor ROI. The latter is essential because high similarity can be achieved in the whole breast region, even if the tumor ROI is poorly synthesized. The performance measures of the whole breast were measured from the entire image (i.e., full field of view [FOV]) and thus it includes contributions from the black background (roughly 1/4 of the FOV). However, performance measures of the tumor ROI were measured from $64\times 64$ cropped images centered on the tumor centroid. We measured the normalized mean squared error (NRMSE), Pearson cross-correlation coefficients (CC), and peak signal-to-noise ratio (PSNR) and structural similarity index map (SSIM) [28], [29], [30]. In the qualitative analysis, we plotted the intensity profiles along the vertical direction of the tumor center so that the intensity trends and details in the target ceT1 image and the synthetic image could be compared. In addition, we generated the error maps by subtracting the synthesized images from the target images for qualitative assessment. The external validation cohort was evaluated using the same four metrics. We did not train on the external validation cohort and simply applied the learned model to the validation cohort to demonstrate the robustness of our model.

### E. Verification of the Effectiveness of Synthesized Image with an Independent Segmentation Task

Other studies suggested that the synthesized images of GAN can be used to improve other downstream tasks [31], [32], [33]. Motivated by these, we conducted an independent segmentation task, separate from the segmentation of our main method, to demonstrate the potential usefulness of the synthesized images in two ways. The first was to evaluate the usefulness of the synthesized ceT1 images compared to the preT1 and ceT1 images. Second, we evaluated the augmentation effects of synthetic ceT1 by training with both target and synthetic T1 images.

An independent segmentation network consisting of the standard 2D U-Net with four down/up sampling layers and was learned using the loss summing the BCE loss and dice loss. This was trained using 2,233 ceT1 images, and 583 images from target ceT1 and synthetic ceT1 images were tested. For the mixed training, the ratio between real ceT1 and synthetic ceT1 data was 1:1. Smaller tumors (less than 3.6 cm^2^) were excluded from evaluation. The segmentation results were compared with target tumor ROIs in terms of BCE, dice similarity coefficient (DSC), and average symmetric surface distance (ASSD) [30], [34].

## III. Results

To evaluate the various models, we compared the results of TSGAN with those of four GAN models: Pix2Pix, ResGAN, EaGAN, and TSGAN-Box.

### A. Quantitative Analysis

Table 1 shows the results of the four evaluation metrics in the test set for both the whole breast and tumor ROIs for various models. Each entry has an average performance metric, followed by the standard deviation in parentheses. We compared TSGAN with other methods using permutation tests and noted entries with p-values less than 0.001 with asterisks (Table 1). Our TSGAN showed the best overall performance for both the whole breast region and tumor ROI. It was more difficult to synthesize the tumor ROI than the entire breast region. Nevertheless, our model showed improved performance in tumor ROIs compared to other models. For the SSIM, a performance difference of approximately 0.06 was present between ours and Pix2Pix. The same trend was observed in all the other models and several evaluation metrics. TSGAN-Box performed slightly worse than TSGAN but was better than other baseline methods (Pix2Pix, ResGAN, and EaGAN).

### B. Qualitative Analysis

**FIGURE 5.**
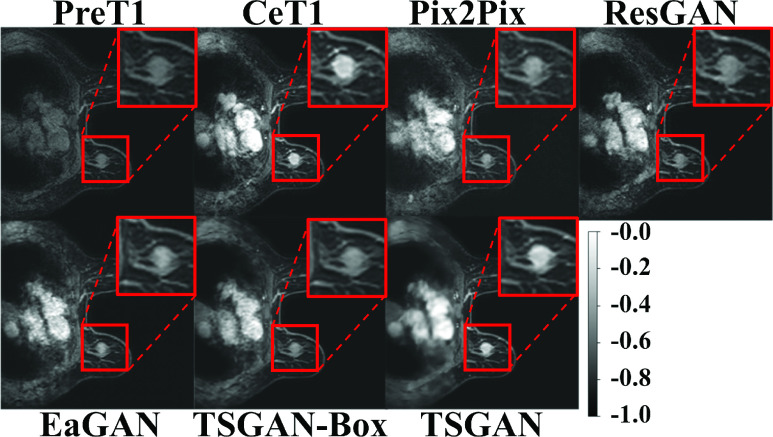
An example of the preT1, ceT1, and synthetic ceT1 images for qualitative analysis. The top two rows visualized the whole breast and the magnified tumor region (red box) using a normalized range between −1 to 0. The first row was represented preT1 (input), ground truth ceT1 (target), and synthesized ceT1 images from Pix2Pix and ResGAN. The second row was the synthesized ceT1 images from EaGAN, TSGAN-Box and TSGAN.

Fig. 5 shows example images and edge maps of the input preT1, target ceT1, and synthesized ceT1 images from the Pix2Pix, ResGAN, EaGAN, TSGAN-Box and the proposed TSGAN. The TSGAN synthesized the other chest anatomical structures and edges (e.g., large circular artery) and enhanced various tissue types better than the baseline models. In particular, for the tumor region, our model generated compact contrast-enhanced patterns better than other methods. The tumor region is characterized by a distinct morphology, enhancement pattern, and texture compared to the surrounding normal tissue. Thus, it was difficult to learn the diverse characteristics of the tumor in other models that synthesize the entire image, not focusing on the tumor. Relatively, our model could concentrate on synthesizing a tumor region by the segmentor and LD. The four evaluation metrics for the cases presented in Fig. 5 are listed in Appendix Table 6, confirming that our model generated the best image.

**TABLE 6:** Quantitative Analysis of Fig. 5. Bold Fond Denotes the Best Performance Among Comparison

	Whole Breast Region			
	NRMSE( $\downarrow$)	PSNR( $\uparrow$)	SSIM( $\uparrow$)	CC( $\uparrow$)
Pix2Pix	30.127	51.836	0.882	0.830
ResGAN	30.162	51.726	0.874	0.810
EaGAN	26.614	52.754	0.901	0.863
TSGAN-Box	26.096	52.885	0.902	0.864
TSGAN	23.832	54.962	0.926	0.907
	Tumor ROI			
Pix2Pix	29.235	69.269	0.815	0.837
ResGAN	31.499	68.346	0.804	0.818
EaGAN	28.148	69.488	0.842	0.869
TSGAN-Box	28.767	69.699	0.835	0.871
TSGAN	22.179	75.906	0.914	0.910

**FIGURE 6.**
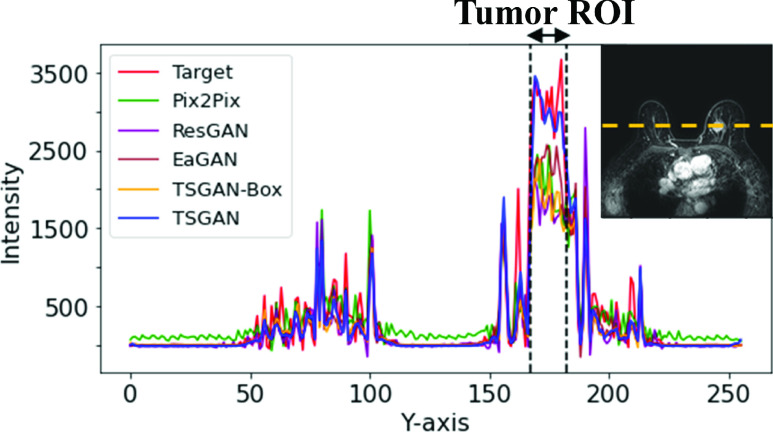
Representative intensity profiles. The intensity changes of the ground truth ceT1 image (“Target”), synthesized ceT1 images from Pix2Pix, ResGAN, EaGAN, TSGAN-Box, and TSGAN are given as red, green, purple, brown, yellow, and blue lines, respectively.

Fig. 6 shows the intensity profiles along the yellow dotted line passing through the center of the tumor. The red line is the pixel intensity profile of target ceT1(ground truth). The blue line was the intensity profile of the synthesized image from the TSGAN model, and it closely followed the tumor intensity profile of the target ceT1 image in the whole breast region. The other four lines (green, purple, sky blue, and yellow) are for comparison models. The region between the black dotted lines indicated the intensity profile of the tumor ROI. Within the tumor extent, the other four lines were further away from the red line than the blue line, which indicates that our model could mimic detailed intensity values of transition of tumor ROI better. We also inspected the green line in the background region and corroborated it to have higher noise levels compared to the noise in our findings.

### C. External Validation

We validated our model and compared it with other models in an external validation cohort. The results showed a similar trend to that of the internal test set. Our TSGAN model attained the best results in both quantitative and qualitative analyses.

Table 2 shows the quantitative results of the four evaluation metrics for both the whole breast and tumor ROI for the external validation cohort. We compared TSGAN with other methods using permutation tests and noted entries with p-values less than 0.001 with asterisks (Table 2). TSGAN showed the best performance in all metrics for the whole breast and tumor ROI. Stable results were confirmed with a higher performance than the other models in most evaluations. However, a shift in the data distribution was observed (e.g., MRI equipment, sequence, and noise). The evaluation metrics for the external validation were numerically lower than those from the test set of the internal cohort.

**FIGURE 7.**
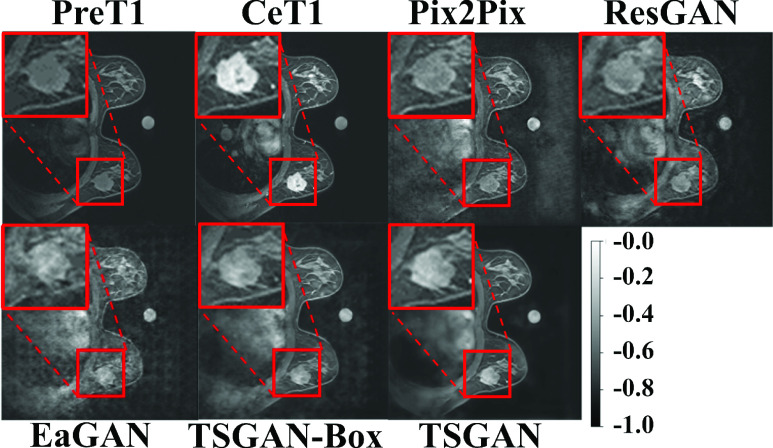
An example of the preT1, ceT1, and synthetic ceT1 images for the external validation cohort for qualitative analysis. The top two rows visualized the whole breast and the magnified tumor region (red box) using a normalized range between -1 to 0. The first row was represented preT1 (input), ground truth ceT1 (target), and synthesized ceT1 images from Pix2Pix and ResGAN. The second row was the synthesized ceT1 images from EaGAN, TSGAN-Box, and TSGAN.

**FIGURE 8.**
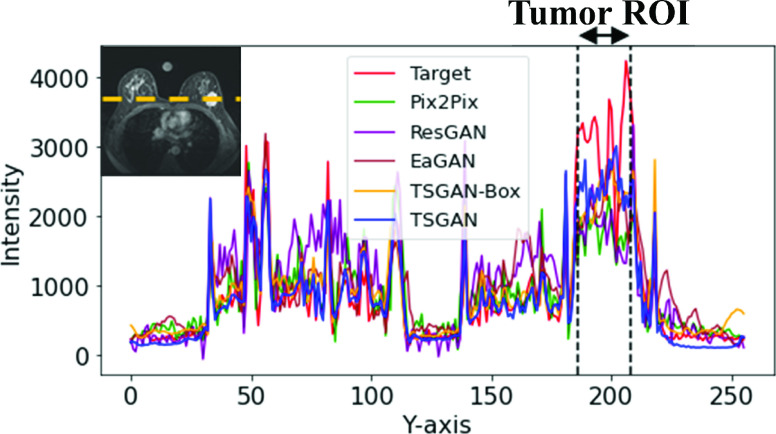
Representative intensity profile for the external validation cohort. The intensity changes of the ground truth ceT1 image (“Target”), synthesized ceT1 images from Pix2Pix, ResGAN, EaGAN, TSGAN-Box, and TSGAN are given as red, green, purple, brown, yellow, and blue lines, respectively.

**FIGURE 9.**
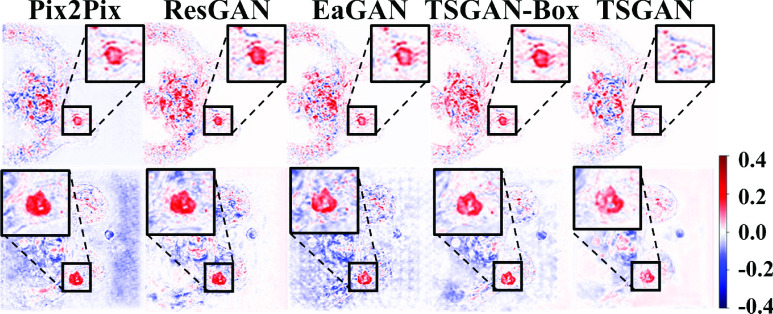
Error maps between ceT1(target) image and various synthetic ceT1 images from Pix2Pix, ResGAN, EaGAN, and TSGAN-Box for qualitative analysis. The first row showed the error maps from a representative case (as shown in Fig 5.) for the internal test dataset and the second row showed the error maps from a representative case (as shown in Fig 7.) for the external validation cohort.

Fig. 7 shows example images of the input preT1, target ceT1, and synthesized ceT1 images from various methods obtained from the external validation cohort. Because noise was absent during training, the synthesis of the chest region was blurry. Nevertheless, the tumor ROI from our method showed enhancement patterns better than the four models (Appendix Table 7). Fig. 8 depicts representative intensity profiles in the external validation cohort. Similar to the results of the internal test set, the synthetic image from our model matched the ground truth tumor ROI better than the images from other models. Fig. 9 shows the representative error maps between ceT1(target) image and various synthetic ceT1 images from Figs 5 and 7.

**TABLE 7:** Quantitative Analysis of Fig. 7. Bold Fond Denotes the Best Performance Among Comparison

	Whole Breast Region			
	NRMSE( $\downarrow$)	PSNR( $\uparrow$)	SSIM( $\uparrow$)	CC( $\uparrow$)
Pix2Pix	35.173	47.979	0.776	0.466
ResGAN	27.245	53.069	0.839	0.452
EaGAN	31.626	49.051	0.829	0.614
TSGAN-Box	24.875	54.608	0.876	0.731
TSGAN	20.024	58.771	0.906	0.781
	Tumor ROI			
Pix2Pix	30.961	63.922	0.713	0.768
ResGAN	31.626	49.051	0.829	0.614
EaGAN	31.469	63.429	0.701	0.765
TSGAN-Box	25.598	67.557	0.802	0.879
TSGAN	22.253	72.766	0.868	0.895

### D. Validity of the Synthesized Image in the Segmentation Task

Table 3 shows the segmentation performance of our independent segmentation task. A previous study for segmentation reported a DSC of 0.67(0.26) in the mean(standard deviation) format using breast ceT1 images and 2D U-Net [35]. Similarly, when the model trained with the ceT1 image was tested with the ceT1 image, it showed a DSC of 0.656(0.237). When tested with the preT1 image, the DSC was 0.166(0.258). However, when tested with the synthetic ceT1 image, the DSC was 0.622(0.270), which was much higher than that obtained using the preT1 image and comparable to that obtained using the target ceT1 image. The BCE and ASSD of testing on the synthetic ceT1 were slightly worse but comparable to those of the target ceT1 images.

In addition, training with both synthetic ceT1 and target ceT1 images resulted in better segmentation performance for all three evaluation metrics. When tested with the ceT1 image, the DSC increased by 0.025, the BCE decreased by 0.002, and the ASSD decreased by 0.039 compared to the training with only ceT1. In summary, we confirmed that the synthetic ceT1 images generated by our model could be useful for segmentation tasks that require ceT1 images. Representative segmentation results of Table 3 comparing various training and test configurations are given in the appendix confirming benefits of mixed training (Appendix Fig. 10).

### E. Ablation Study

To evaluate the performance gains of various components, we started with the residual GAN equivalent to Pix2Pix with residual modules. The addition to the SN, LD, edge maps, and segmentor boosted the synthesis performance, as shown in Table 4. We demonstrated that each element of our TSGAN model contributed to performance gains in its role in synthesizing the whole breast ceT1 image.

## IV. Discussion

We proposed a novel approach of TSGAN to synthesize breast ceT1 images from preT1 images in an end-to-end fashion. Our TSGAN focused on the tumor region critical in breast DCE MRI by adding a segmentation network and LD to focus on the tumor region. Simultaneously, we overcame the limitations of the classical GAN to achieve stable learning by adapting residual learning and SN. In addition, the attention module, edge maps, and feature matching helped capture the complex nonlinear mapping from preT1 to ceT1 images. The four similarity evaluation metrics were measured over both the internal and external validation cohorts in the whole breast region and tumor ROI. Our model synthesized the most authentic-like ceT1 images compared to the other GAN models.

Residual learning is known for providing stable performance in neural networks, and this facilitates stable training in our generator. SN is another technique for stable learning, which was applied to three networks (i.e., generator, GD, and LD) in our method. The self-attention module encoded important local information from the feature maps for the synthesis task. The edge map successfully expanded the generator to consider the anatomical critical structure and shape characteristics in addition to pixel intensities in two discriminators (GD and LD). In addition, we discovered that two discriminators with feature matching induced the generator to synthesize more realistic images for the whole breast and tumor ROI. Most importantly, the segmentor and LD guided the generator to seek detailed enhancement patterns and feature representation of the tumor region. Therefore, the generator of our model learned to resemble the distribution of synthetic ceT1 images to that of target ceT1 images. The synthetic images of our model showed better results compared to the other models in both quantitative and qualitative analyses in both the whole breast and tumor ROI. In addition, we observed that our model generated enhancement patterns better than several other models, even in the external validation cohort. The results of the independent segmentation task demonstrated the validity and usefulness of the synthetic ceT1 images.

Existing GAN studies on breast imaging (e.g., mammography and MRI) have been reported [36], [37]. Modanwal et al. proposed a normalization method for breast MRI using a cycle-consistent GAN [38]. Korkinof et al. proposed the MammoGAN for the high-resolution synthesis of realistic full-field digital mammograms [39]. The two studies focused on the entire breast and not the tumor region. Some studies have noted the importance of the tumor region, similar to our approach [40]. Wu et al. demonstrated the improvement of mammogram classification by synthesizing breast lesions using contextual GANs [41]. Sun et al. conducted a study of MRI reconstruction focusing on the breast using a deep convolutional generative adversarial network from low-resolution preT1 and ceT1 images to high-resolution preT1 and ceT1 images [42]. These studies focused on the breast or tumor region but required additional manual cropping to remove the chest area or annotation of the tumors. However, typical breast MRI contains the chest region, and our method is easier to apply than other methods because our method does not require manual adjustments.

Vision transformers bring high-performing attention mechanisms from the natural language processing domain to the image domain. It has been increasingly applied to various medical imaging modalities including MRI [43], [44]. One study proposed a framework to synthesize a new modality from an existing set of modalities based on a residual convolution transformer module [45]. It could certainly be applied to our setting to synthesize T1-weighted contrast-enhanced MRI (ceT1) images from pre-contrast T1-weighted MRI (preT1). Such models are typically highly complex and properly training the model requires data-specific pre-training [46], [47], while ours is relatively less complex and does not require pre-training. Even with the increased complexity, pursuing transformers in medical image synthesis is a promising future research direction.

Dalmaz et al. approached the medical image synthesis in the federated learning framework using a robust GAN with latent features specific to individual sites and source-target contrasts [48]. Ozbey et al. synthesized medical images in a GAN framework using a conditional diffusion process to improve robustness [49]. Our TSGAN adopted traditional methods of feature matching and spectral normalization to improve the stability of our GAN. In addition, our segmentor forced the network to focus on the tumor region that would stabilize the overall network since it focused heavily on the small tumor region. We plan on borrowing ideas of robust GAN using the diffusion process for a centralized setting and site-specific modeling for a federated setting.

We constructed a stacked 2D slice model with three slices. We stacked three consecutive axial slices skipping every other slice (e.g., 1, 3, and 5th slice) where the tumor was present as the input and made sure there was no overlap between two sets of stacked slices. The model showed 26.137 (2.512) of NRMSE (standard deviation [STD]), 0.902 (0.025) of SSIM, 52.810 (2.127) of PSNR and 0.899 (0.025) of CC in the internal test dataset for the whole breast. The model showed 24.940 (4.552) of NRMSE (STD), 0.861 (0.060) of SSIM, 73.730 (4.028) of PSNR and 0.857 (0.065) of CC for the tumor ROI. The stacked 2D model performed worse than TSGAN partly because it had fewer samples to train the model. The stacked 2D model was trained with 654 sets of slices which was much smaller than the 2,233 slices used for TSGAN.

Our study has several limitations. First, we used a 2D model using image slices as input and not 3D volumes. DCE MRI is inherently 3D, and thus, a 3D model is a natural choice. However, we could not adopt a full 3D model owing to hardware constraints and sample size issues. If our proposed model is trained using a 3D model, it might result in better performance. This is left for future work. Second, our breast DCE MRI data were derived from mass tumor cases, and we did not consider non-mass enhancement tumors. In the future, we need to explore additional breast tumor types so that the proposed network can be applied to a broad range of breast tumors. Third, our synthesized images were not evaluated with conventional manual evaluations, such as the Breast Imaging-Reporting and Data System (BI-RADS), which is widely used for clinical diagnosis. Such efforts require significant expert resources and are thus left for future work.

## V. Conclusion

In conclusion, we introduced an enhanced GAN to synthesize a realistic ceT1 image by applying the segmentation task and demonstrated the feasibility of ceT1 synthesis focusing on tumor ROIs. Our TSGAN is a neural network and thus once trained it could be applied in a matter of milliseconds for new incoming patients without additional time delay in the existing clinical workflow. Our TSGAN can be easily integrated into the existing clinical workflow in the form of an additional software module to existing image analysis/archiving software. We hope that our method will help patients avoid potentially harmful CAs, resulting in an improved diagnosis and treatment workflow for breast cancer.

## Figures and Tables

**FIGURE 10.**
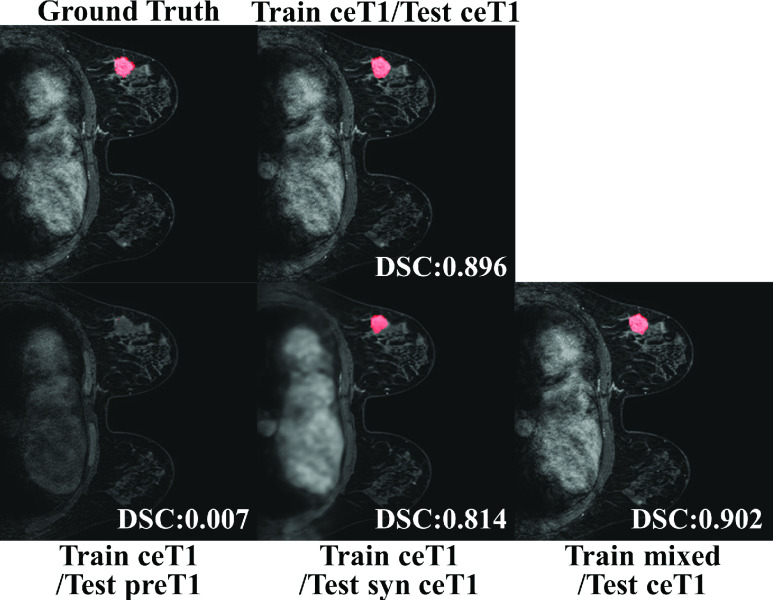
Representative segmentation results of Table 3 comparing various training and test configurations. The first row has the ground truth tumor ROI with ceT1 image (“Ground Truth”) and training and testing with ceT1 (“Train ceT1/Test ceT1”). The second row has results of training with ceT1 and testing with preT1 (“Train ceT1/Test preT1”), training with ceT1 and testing with synthetic ceT1 (“Train ceT1/Test syn ceT1”), and training with mixed data and testing with ceT1 (“Train mixed/Test ceT1”).

## Appendix

### A. Inclusion Criterion

We recruited 440 and 209 patients from the SMC and GH groups, respectively. The inclusion criteria for these cohorts were as follows: (a) preoperative DCE MRI; (b) a final pathological diagnosis of initial unilateral malignancy in invasive breast cancer; and (c) a mass lesion on breast MRI. We excluded patients with DCE MRI according to the following criteria: (a) patients diagnosed with breast cancer by vacuum-assisted or excisional biopsy (n = 42); (b) patients treated with neoadjuvant chemotherapy (n = 92); (c) patients with metastasis or primary malignancy (n = 9); (d) involvement of any resection margin at the final pathology (n = 16); (e) patients who had an invisible case of known breast cancer (n = 21); and (f) inadequate MRI quality for further processing (n = 23). Finally, we included 306 and 140 patients from the SMC and GH groups, respectively, in this study.

### B. Imaging Parameters

For the SMC cohort, MRI scans were acquired using a fat-suppressed 3D DCE sequence. Axial images of both breasts were acquired with the patient in the prone position. Each subject underwent scans from six dynamic phases after the injection of the contrast agent (gadolinium). A 0.1 mmol/kg bolus of gadobutrol (Gadovist; Bayer Healthcare Pharmaceutical, Berlin, Germany) was injected for dynamic contrast enhancement, and a 20 ml saline flush was injected. For GH, MRI scans were acquired using a fat-suppressed 3D DCE sequence. Each scan had a pre-contrast and five post-contrast dynamic series. A 0.1 mmol/kg bolus of gadoterate meglumine (Dotarem; Guerbet and Clariscan, GE Healthcare, USA) was injected, followed by a 20 ml saline flush. Detailed imaging acquisition information for these two cohorts is shown in Appendix Table 5. Nearest-neighbor interpolation was applied to resample the ROIs drawn from the original data to the data with isotropic resolution.

### C. Supplementary Results

See Tables 6 and 7 and Fig. 10.
